# Natural history of inflammation and impaired autophagy in children with Gaucher disease identified by newborn screening

**DOI:** 10.1016/j.ymgmr.2025.101187

**Published:** 2025-01-21

**Authors:** V. Gragnaniello, D. Gueraldi, A. Saracini, D. Velasquez Rivas, C. Cazzorla, L. Salviati, A.B. Burlina

**Affiliations:** a Division of Inherited Metabolic Diseases, Department of Women's and Children's Health, University of Padua, Padua, Italy; b Division of Inherited Metabolic Diseases, Department of Women's and Children's Health, University Hospital of Padua, Padua, Italy; c Clinical Genetics Unit, Department of Women's and Children's Health, University of Padua, Padua, Italy

**Keywords:** Gaucher disease, Inflammation, Autophagy, Newborn screening

## Abstract

**Introduction:**

Gaucher disease is a lysosomal storage disease due to deficiency of glucocerebrosidase, leading to the accumulation of glucosylceramide, particularly in macrophages. In addition to storage, secondary abnormalities such as inflammation, cellular stress, and impaired autophagy may contribute to the disease pathogenesis. The onset and course of progression of these secondary abnormalities remains unclear. Owing to the increasingly widespread newborn screening programs, diagnosis can be made at a presymptomatic stage. Understanding the early natural course of the disease is important for optimal monitoring and management of such at-risk individuals.

The aim of our study is to investigate secondary abnormalities in very young children with type 1 Gaucher disease identified through neonatal screening.

**Materials and methods:**

We enrolled five children (<4 years old) with type I Gaucher disease in a presymptomatic stage and not receiving therapy. We assessed plasma cytokine profiles (TNFα, IL1β, and IL6 by ELISA), activation of pro-inflammatory p38 mitogen-activated protein kinase (MAPK) and the abundance of LC3-II as indicator of autophagic flux, by immunoblotting.

**Results:**

All subjects exhibited elevated TNFα (mean 21.74 μmol/L, SD 37.48, range 2.37–88.72 μmol/L). The other cytokines analyzed were within normal range. Cellular stress (activation of p38) was present in the child with higher glucosylsphingosine (GluSph) accumulation. Additionally, all subjects showed a significant reduction in LC3-II (mean 88 %, SD 9 %, range 77–98 %), indicating reduced autophagic flux.

**Discussion:**

We have identified the presence of inflammation with inhibition of autophagic flux in presymptomatic young children with a genetically confirmed high-risk of developing Gaucher disease. These findings contribute insights into the early course of Gaucher disease and support the management of at-risk individuals identified by newborn screening. Therapeutic interventions including specific enzyme replacement or other means to address inflammation or autophagy could delay or prevent the onset of symptomatic disease and consequential disability. Further clinical studies are warranted to explore these possibilities.

## 1 Introduction

Gaucher disease (GD) is an autosomal recessive disorder caused by variants in the *GBA1* gene, which encodes the lysosomal enzyme glucocerebrosidase (GCase). GCase catalyzes the hydrolysis of glucosylceramide (GluCer) to ceramide and glucose, and its deficiency results in the accumulation of GluCer within lysosomes [1]. The hallmark cell type affected in GD is the lipid-laden macrophage known as the Gaucher cell, which accumulates predominantly in the liver, spleen, bone marrow, and lung, leading to organ enlargement and progressive dysfunction [2,3].

GD is clinically classified based on central nervous system involvement. GD1, the most common form (approximately 90 % of GD cases), is characterized by chronic, non-neuropathic symptoms including visceral and skeletal manifestations (splenomegaly, hepatomegaly, and bone abnormalities) and hematologic abnormalities (pancytopenia) [4,5]. GD2 and GD3 represent acute and chronic forms of neurovisceral GD, respectively [5,6].

Despite GCase deficiency and the associated GluCer accumulation being the primary cause of GD, many clinical features cannot be fully explained by the mass of undegraded GluCer within macrophages. Recent research has focused on exploring secondary abnormalities that may contribute to disease pathogenesis, including inflammation, cellular stress, and the autophagy pathway. It has been proposed that chronic macrophage activation may lead to persistent immune system stimulation and systemic inflammation, impacting tissues and cells beyond those directly affected by GluCer accumulation [7,8]. This may account for phenomena such as increased prevalence of hypergammaglobulinemia, monoclonal gammopathies, elevated risk of hematologic malignancies, bone disease, and potentially central nervous system involvement in neuronopathic forms of GD [[9], [10], [11]]. Additionally, since the extent of visceromegaly exceeds the mass of GluCer storage by two orders of magnitude, a florid inflammatory tissue reaction is a major component of the systemic disease [12].

Understanding the natural course of these secondary abnormalities is crucial, especially with the expanding implementation of neonatal screening for lysosomal diseases, including GD, over the past two decades. Neonatal screening enables early identification of individuals with high risk of developing GD, including both early-onset and later-onset cases [13]. Currently, conventional therapies such as enzyme replacement therapy and substrate inhibitors are not recommended before clinical symptoms appear [[14], [15], [16]]. However, while it is known that GluCer accumulation begins prenatally, allowing the use of Glusph as a second-tier test in neonatal screening [17], the timing and impact of the secondary abnormalities in early disease progression remain unclear. Existing studies often involve heterogeneous populations at various disease stages and under different treatment regimens, and it is often unclear whether the reported abnormalities are the cause or the consequence of the damage. Furthermore, research on intracellular stress and autophagy pathways frequently relies on animal models or in vitro studies, which may not fully replicate in vivo anomalies.

Here we investigated inflammatory and secondary pathways of cellular stress and autophagy in peripheral blood cells of presymptomatic children with GD1 identified through neonatal screening, implemented in Northeast Italy since 2015 [13,18].

## 2 Materials and methods

### 2.1 Study population

Children identified with non-neuronopathic Gaucher disease (GD1) by neonatal screening and followed at the Inherited Metabolic Diseases Unit of the University Hospital of Padua were recruited. Inclusion criteria: age < 4 years; a confirmed diagnosis of GD1 by biochemical (GCase activity in lymphocytes, plasma GlcSph by MS/MS) and molecular (*GBA1* gene) testing; absence of signs and symptoms of disease (including bone pain); no cytopenia, liver enzyme activities within the healthy reference range, absence of hepatosplenomegaly (confirmed by abdominal ultrasonography). Exclusion criteria comprised presence of any symptoms or physical signs of disease, concomitant diseases or the use of drugs or nutritional supplements. In particular, a complete physical examination, along with the evaluation of the blood count and inflammation markers (CRP), allowed for the exclusion of concomitant infectious diseases.

All caregivers provided informed consent for participation in the study, conducted in accordance with the principles of the Declaration of Helsinki. The study was approved by the Research Ethics Committee of the University Hospital of Padua (n. 39,776, 5 June 2024).

### 2.2 Sample collection and analysis

Blood samples were collected in heparinized vials via venous puncture during outpatient follow-up visits. Plasma was used to measure GlcSph by LC-MS/MS and cytokines TNFα, IL-1β, and IL-6 by ELISA. Normal values were determined by previous studies [19]. The remaining cell pellet was processed to isolate peripheral blood mononuclear cells (PBMCs), which were assayed by immunoblotting to determine the P-p38 (the activated form of p38 MAPK, MW 42 kDa) and LC3 levels (LC3-I MW 16 kDa, LC3-II MW 14 kDa), as we previously described [20]. Four healthy control samples for immunoblotting were obtained from residual specimens of subjects (<4 years) screened for unrelated inherited conditions and in whom no abnormalities were found.

### 2.3 Gene nomenclature

We followed the standard naming convention of the Human Genome Variation Society for the *GBA1* gene, such that methionine encoded by the translation initiation codon was designed as position 1 in amino acid numbering, by contrast with common *GBA1* variant naming, which omits amino acid residues of the *GBA1* signal peptide.

### 2.4 Statistical analysis

Results are presented as mean, standard deviation, and range.

## 3 Results

### 3.1 Study population

**Fig. 1:**
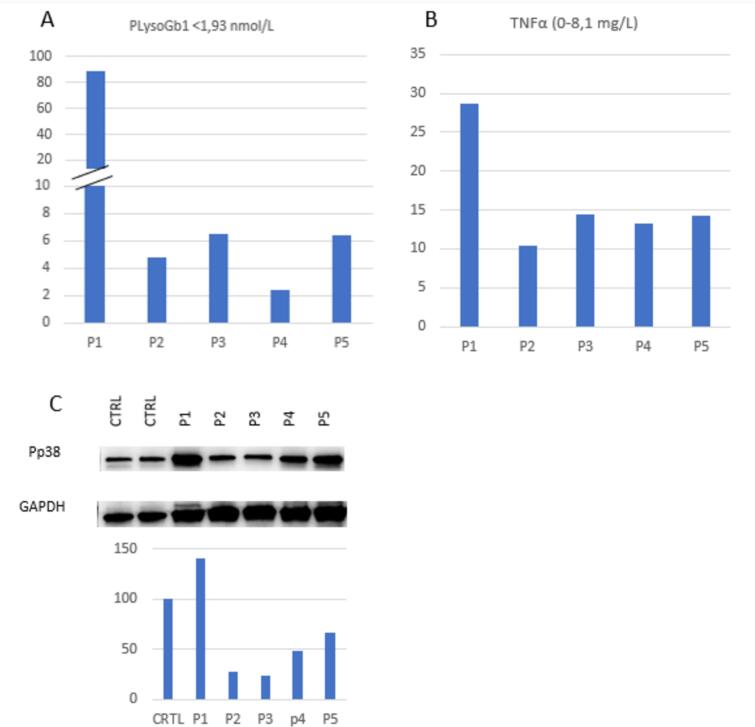
Plasma GlcSph (a), TNFα (b) and PBMC P-p38 levels (c) in studied subjects.

Our neonatal screening program identified 16 children (12 males, 4 females) at risk for the development of Gaucher disease between 2015 and 2023 [13]. Of these, five subjects (4 males, 1 female) met the inclusion and exclusion criteria and were enrolled [Table 1]. Each child carried two *GBA1* variants, of which at least one p.Asn409Ser allele (c.1226 A > G, formely p.Asn370Ser) and was diagnosed with GD1. At the time of enrollment, all children exhibited plasma GluSph levels above the cut-off. The mean was 21.7 μmol/L (SD 37.5, range 2.37–88.7 μmol/L, nv 1.93 μmol/L, *n* = 5) (Fig. 1a). The lowest value was found in patient S4, carrying the variants p.Asn409Ser and p.Thr408Met, the latter classified as of uncertain significance, according to the ClinVar database [https://www.ncbi.nlm.nih.gov/clinvar/RCV001249086.1/; last access 6 Dec 2024].

**Table 1:** Study population: clinical and biochemical parameters at diagnosis (newborn screening) and at enrollment.

	Newborn screening		Confirmatory tests						Last evaluation			
	Gcase on DBS (μM/h)*	LysoGb1 on DBS (nv 5.64–33.31)	Plasma LysoGB1 (nv < 1.93 μmol/L)	Gene Variant 1	Gene variant 2	Protein variant 1	Protein variant 2	Clinical manifestations	Age at last evaluation	Clinical manifestations	Plasma LysoGb1 (nv < 1.93 μmol/L)	Therapy
S1	0.5	231.7	23.6	c.1226 A > G	c.880 T > G + 1342G > C	Asn409Ser	His294Gln+ Asp448His	No	4 yrs.	No	88.72	No
S2	1.07	116.16	12.73	c.1226 A > G	c.680 A > G	Asn409Ser	Asn227Ser	No	4 yrs	No	4.75	No
S3	2.3	107.2	8.14	c.1226 A > G	c.1226 A > G	Asn409Ser	Asn409Ser	No	3 yrs	No	6.49	No
S4	1.42	53	3.74	c.1226 A > G	c.1223C > T	Asn409Ser	Thr408Met	No	3.5 yrs	No	2.37	No
S5	1.71	52.25	2.67	c.1226 A > G	c.1093G > A	Asn409Ser	Glu365Lys	No	3 yrs	No	6.39	No

* The cutoff is set at 0.2 Multiple of Median. It is recalculated monthly, due to seasonal changes in temperature and humidity during transport [13,18].

### 3.2 Inflammation

TNFα levels were elevated in all enrolled children. The mean TNFα value was 16.2 mg/L (SD 7.1, range 10.4–28.6, nv <8.1 mg/L). The highest TNFα level was observed in the subject S1, who also had the highest Glusph level (88.7 μmol/L) (Fig. 1b). The other cytokines analyzed were within normal range.

Cytokines, particularly TNFα, regulate various intracellular pathways, including the MAPK (mitogen-activated protein kinase) pathways. We investigated the activation of p38 by evaluating the levels of its phosphorylated form, P-p38.

The level of P-p38 in S1, who exhibited the highest levels of storage and inflammation (GluSph and TNFα, respectively), was 1.5 times higher than controls (Fig. 1c).

### 3.3 Autophagy

Closely related to inflammation and cellular stress is the autophagy pathway. To assess cellular autophagic flux, we performed LC3 immunoblot analysis. The level of LC3-II correlates with autophagosome abundance, making it a reliable indicator of autophagic flux.

**Fig. 2:**
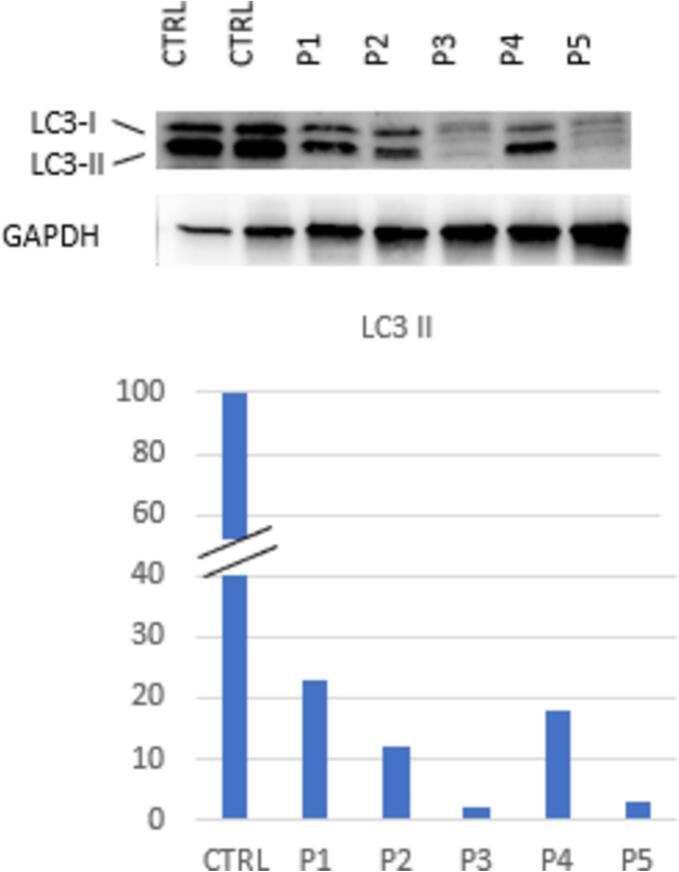
LC3 levels in studied subjects. Endogenous LC3 is detected as two bands: LC3-I, a cytosolic form (16 kDa), and LC3-II, which is lipidated and associated with isolation membranes and autophagosomes (14 kDa) until fusion with lysosomes.

Enrolled children exhibited an average reduction of 88 % (SD 9 %, range 98–77 %) in LC3-II levels compared with control subjects, indicating a significant inhibition of autophagy (Fig. 2).

## 4 Discussion

Here we show that signs of inflammation, cellular stress, and altered autophagy are present in very young asymptomatic children with the enzymatic, biochemical and genetic characteristics of GD. The study included a homogeneous population comprised five pediatric subjects, identified through a rigorous newborn screening procedure. At enrollment, all were asymptomatic and aged between 3 and 4 years.

To explore the presence of an inflammatory status, we evaluated the plasma cytokine profile. All these children had increased plasma TNFα concentrations, which we attribute to constitutively activated macrophages already present in the early stages of the disease; it is notable that the highest TNF-α was measured in the child who also showed the greatest concentration of GlcSph. The levels of IL1β and IL6 were within the normal range.

Previous studies have reported elevated circulating TNFα and other cytokines such as IL-1 and IL-6 in patients with GD, although results have been occasionally conflicting. For instance, Michelakakis et al. examined plasma TNFα concentrations in patients with various types of GD, noting highest concentrations in those with neuronopathic forms, while individuals with GD1 exhibited a wide range of TNFα values, varying from normal to 2.5 times the upper control limit [21]. Other studies have also indicated elevated circulating TNFα, often associated with disease severity, yet independent of residual white blood cell or fibroblast GCase and chitotriosidase activities [[21], [22], [23], [24], [25], [26], [27]]. The increase in IL1β and IL6 is reported variably only in some studies [22,26]. These studies are limited by heterogeneous study populations (GD1 vs. GD3; splenectomized vs. non-splenectomized, treated vs. untreated, with or without concurrent conditions such as malignancies, monoclonal gammopathy, active infections). The pathological release of serum inflammatory cytokines in asymptomatic subjects identified at birth with a high probability of developing full-blown Gaucher disease has not hitherto been explored in depth.

Our data show that plasma TNFα concentrations are elevated from the early stages of GD and correlate with an excess of a primary glycosphingolipid substrate indicated by elevated glucosylsphingosine, supporting the hypothesis that the pathological lysosomal storage environment activates acid ceramidase to generate this lysosphingolipid and consequentially triggers cytokine release by the Gaucher cell. This finding provides crucial insights into the early pathogenesis of GD1: not only does GlcCer accumulation begin in fetal development, but cytokine dysregulation, known to mediate damage in later stages, is also initiated in early childhood.

The absence of increase in IL1β and IL6 is not surprising. The isolated increase in TNFα reflects the central role of activated macrophages in this pathology and the chronic, low-grade nature of the inflammation. The pathways of IL1b and IL6 may require additional or specific stimuli (e.g., acute damage or inflammasome activation) that are not always present in Gaucher disease, especially in the early stages.

Cytokines can have multiple systemic effects, particularly TNFα is a pleiotropic cytokine that regulates two major signaling pathways, NF-kB and MAPK. Nuclear factor kappa B (NF-kB) governs the expression of genes encoding various proinflammatory factors, perpetuating the inflammatory stimulus [28].

Regarding the MAPK pathway, we investigated the activation of the proinflammatory kinase p38. Previous in vivo studies have demonstrated p38 activation in GD mice, showing increased P-p38 levels in brain tissues of neuronopathic mice and other affected organs such as liver and lung across all types of GD [29]. However, whether p38 is similarly activated in human GD has not been well-established. Our study revealed increased P-p38 level in subject S1, who also exhibited the greatest concentration of GluSph and TNFα in the study group. We contend that this consistent with the hypothesis that p38 activation is driven by both glucosylceramide accumulation and inadequate ceramide production due to GCase deficiency [30]. Overactive p38 activates various inflammatory mediators, including TNF-α, prostaglandins, and reactive oxygen species (ROS), amplifying the inflammatory milieu [31]. Its activation in subject S1 aligns with the observed inflammatory state and is an indication of cellular stress at an early phase of the illness.

Another objective of our study was to evaluate alterations in the autophagy pathway, which may be linked to the observed inflammatory abnormalities. Similar to other lysosomal storage disorders, GlcCer accumulation within lysosomes can disrupt a critical step in autophagy: the fusion of autophagic vacuoles with lysosomes to form autophagolysosomes [32]. The abundance of LC3, analyzed by immunoblotting, can provide insights into these late stages of the autophagic pathway. In vitro studies using a macrophage model [33] and animal models of GD such as the fruit fly, *Drosophila* [34,35] and mice [36,37] have demonstrated increased LC3-II levels, indicating a defect in autophagosome-lysosome fusion and increase in the number of autophagosomes. Contrary to these findings, Western blot analysis in our population revealed significantly decreased levels of LC3-II. The reduction of LC3-II indicates a reduction of the autophagic flux and autophagosome formation rather than a block in their fusion with the lysosome, suggesting impaired constitutive autophagy.

These discrepancies may origin from differences between models, as the observations in vitro and animal models may not fully replicate human conditions. A second possibility is that autophagic deficiency impacts various points of the *endo*-lysosomal network in a stepwise manner, leading to different findings in different stage of the disease. We hypothesize that initially, in a presymptomatic phase, when autophagosome-lysosome fusion is not severely compromised, inhibition of autophagic flux may explain the reduction of LC3-II. Accordingly, several studies have reported mTOR hyperactivation in cellular models of GD [38,39], potentially explaining the inhibition of autophagic flux. Under conditions of nutrient excess, including the accumulation of glycosphingolipids, mTOR phosphorylates and inactivates TFEB, a transcription factor crucial for autophagy regulation. Consistent with this hypothesis, studies have shown reduced TFEB and stability in models of human induced pluripotential stem cells prepared from patients with GD that show lysosomal depletion and impaired autophagy [40]. The reduction of TFEB can lead to an inhibition of autophagic flux with reduced formation of autophagosomes, explaining the reduced levels of LC3-II found in our study. The inhibition of autophagy can contribute to the observed inflammatory status, as defects in inflammasome degradation sustain its constitutive activation, while defective mitophagy perpetuates dysfunctional mitochondria and oxidative stress [33,41].

Limitations of our study include the small sample size due to the rarity of the disease and the strict selection of a presymptomatic population. Additionally, the small cell pellet obtained from residual blood samples of pediatric subjects limited our ability to conduct a comprehensive study of autophagy. Finally, while the selection of subjects carrying at least one p.Asn409Ser allele makes our population more homogeneous, it is known that subjects who are homozygous for this variant can also remain asymptomatic for the disease [[42], [43], [44]]. In particular, subject S4 carried the p.Asn409Ser variant in compound heterozygosity with the p.Thr408Met variant, which is nowadays of uncertain significance [https://www.ncbi.nlm.nih.gov/clinvar/RCV001249086.1/; last access 6 Dec 2024]. However, in our patient the value of GlcSph was above the cutoff (2.37 μmol/L, nv <1.93), not in the normal range showed by heterozygotes [19]. That data maybe explain the signs of inflammation and cellular stress found in the subject. Indeed, it has been reported that GlcSph is not only a marker of the disease but also plays a pathogenic role [45]. Several in vitro and in vivo studies have shown its pro-inflammatory effect [[46], [47], [48]] and the role in the regulation of the autophagy [49]. A long term follow up will be necessary to better understand whether inflammation and autophagy biomarkers may have prognostic and predictive values for the clinical onset of the disease.

To conclude, we have demonstrated for the first time the presence of an inflammatory state and inhibition of autophagy in a homogeneous population of young children with GD who we identified through newborn screening. These biomarkers could prove useful in clinical practice for a more complete and non-invasive characterization of individuals at an asymptomatic phase of the illness to which they are prone. Moreover, the described pathways represent a potential therapeutic target to interrupt or partially arrest the pathogenic processes that cause the onset of frank disease - perhaps even distinct from the more invasive and costly use of enzyme therapy in very young children. In particular, the use of anti-inflammatory drugs, already reported in symptomatic GD patients [12] and drugs targeting pathways such as p38, already utilized in autoinflammatory conditions like rheumatoid arthritis and Crohn's disease [50,51], may find application in GD. Similarly, the autophagy pathway and its regulator, the mTOR complex, present potential therapeutic targets [32]. We contend that further research should focus on investigating the efficacy and safety of such therapeutic strategies in more comprehensive in vitro and in vivo models: this would contribute to understanding the evolution of GD and may improve health outcomes before irreversible manifestations are established.

## Funding

Not applicable.

## CRediT authorship contribution statement

**V. Gragnaniello:** Writing – original draft, Validation, Project administration, Methodology, Investigation, Formal analysis, Data curation, Conceptualization. **D. Gueraldi:** Investigation. **A. Saracini:** Investigation. **D. Velasquez Rivas:** Methodology, Investigation. **C. Cazzorla:** Investigation. **L. Salviati:** Methodology, Investigation. **A.B. Burlina:** Writing – review & editing, Validation, Supervision, Resources, Project administration, Conceptualization.

## Declaration of competing interest

The authors declare no conflict of interest.

## Data Availability

Data will be made available on request.
